# Comparative evaluation of atom mapping algorithms for balanced metabolic reactions: application to Recon 3D 

**DOI:** 10.1186/s13321-017-0223-1

**Published:** 2017-06-14

**Authors:** German A. Preciat Gonzalez, Lemmer R. P. El Assal, Alberto Noronha, Ines Thiele, Hulda S. Haraldsdóttir, Ronan M. T. Fleming

**Affiliations:** Luxembourg Centre for Systems Biomedicine, University of Luxembourg, 6, avenue du Swing, 4367 Belvaux, Luxembourg

**Keywords:** Atom mapping, Metabolic network reconstruction, Automation, RDT, DREAM, AutoMapper, CLCA, MWED, ICMAP, Recon 3D

## Abstract

The mechanism of each chemical reaction in a metabolic network can be represented as a set of atom mappings, each of which relates an atom in a substrate metabolite to an atom of the same element in a product metabolite. Genome-scale metabolic network reconstructions typically represent biochemistry at the level of reaction stoichiometry. However, a more detailed representation at the underlying level of atom mappings opens the possibility for a broader range of biological, biomedical and biotechnological applications than with stoichiometry alone. Complete manual acquisition of atom mapping data for a genome-scale metabolic network is a laborious process. However, many algorithms exist to predict atom mappings. How do their predictions compare to each other and to manually curated atom mappings? For more than four thousand metabolic reactions in the latest human metabolic reconstruction, Recon 3D, we compared the atom mappings predicted by six atom mapping algorithms. We also compared these predictions to those obtained by manual curation of atom mappings for over five hundred reactions distributed among all top level Enzyme Commission number classes. Five of the evaluated algorithms had similarly high prediction accuracy of over 91% when compared to manually curated atom mapped reactions. On average, the accuracy of the prediction was highest for reactions catalysed by oxidoreductases and lowest for reactions catalysed by ligases. In addition to prediction accuracy, the algorithms were evaluated on their accessibility, their advanced features, such as the ability to identify equivalent atoms, and their ability to map hydrogen atoms. In addition to prediction accuracy, we found that software accessibility and advanced features were fundamental to the selection of an atom mapping algorithm in practice.

**Electronic supplementary material:**

The online version of this article (doi:10.1186/s13321-017-0223-1) contains supplementary material, which is available to authorized users.

## Background

**Fig. 1:**
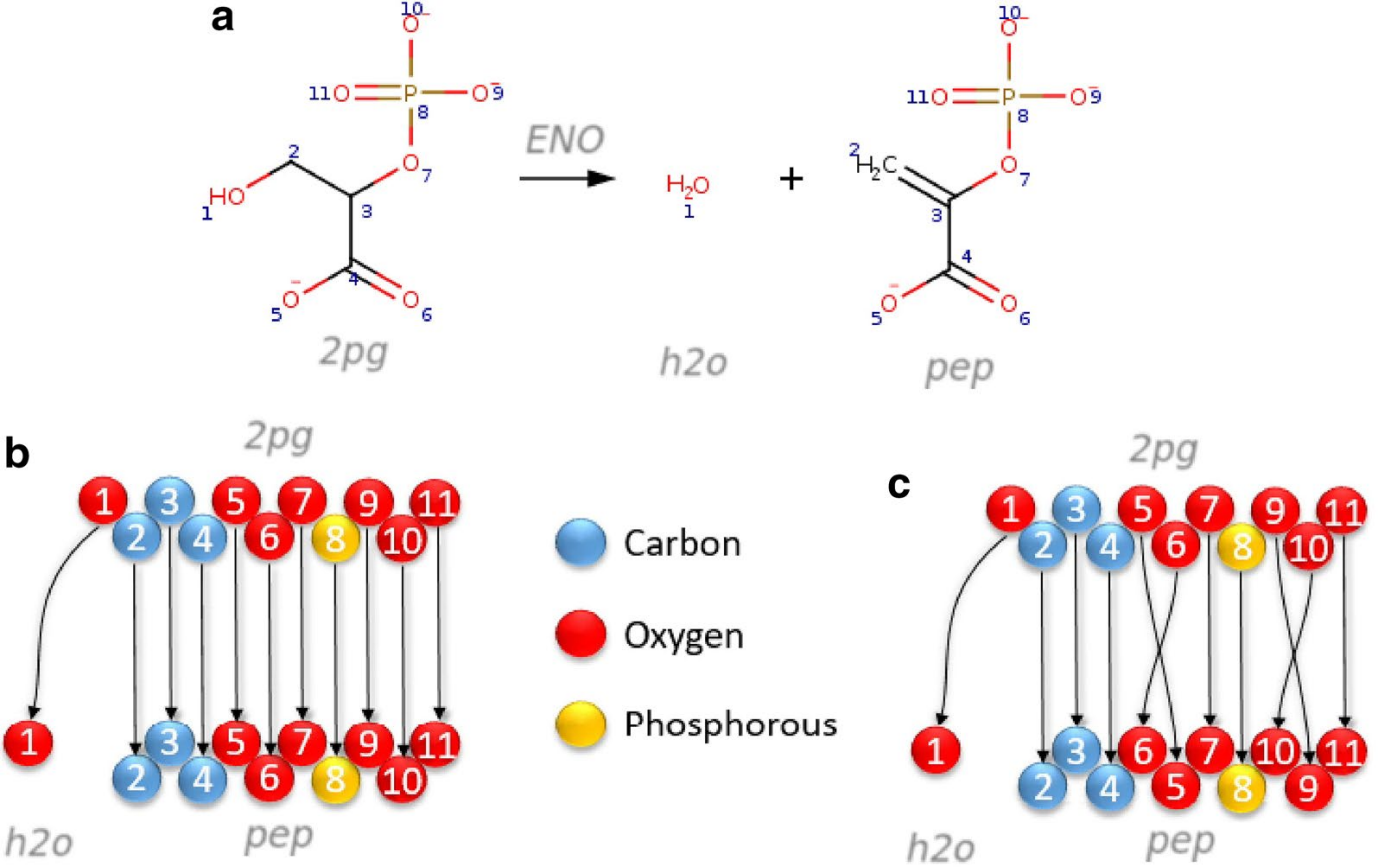
An atom mapping for the enolase reaction. **a** Enolase (VMH ID: ENO) catalyses the hydrolysis of 2-phosphoglycerate (VMH ID: 2pg) to produce phosphoenolpyruvate (VMH ID: pep) and water (VMH ID: h2o). The atoms of the substrate are assigned with a mapping number that matches only with one atom of the same element in the product molecules; this representation describes the reaction mechanism. **b**, **c** A graphical representation of two possible atom mappings for the enolase reaction. Nodes (*circles*) represent atoms. Atoms can be matched to metabolite structures in (**a**) on their metabolite identifiers, *colours* and *numbers*. Directed edges (*arrows*) represent atom transitions. All hydrogen atoms are omitted to simplify the figure. Since oxygen atoms *5*, and *6* and *9*, *10*, and *11* are chemically equivalent twelve accurate atom mappings could be predicted for this reaction

In every biochemical reaction, the total number of atoms of each element in all substrates is equal to that in all products. An atom mapping is a one-to-one correspondence (bijection) between an atom in a substrate and an atom in a product. An instance of a chemical reaction may be represented by a set of atom mappings, with one atom mapping between each substrate and product atom. Together, a set of atom mappings for a chemical reaction specify key aspects of the reaction mechanism, e.g., chemical bond change, breakage, and formation. A single chemical reaction can admit multiple chemically equivalent atom mappings when chemically equivalent atoms are present in a substrate, a product, or both. Therefore, each chemical reaction can be represented by one set, or multiple chemically equivalent sets, of atom mappings, each of which may be interpreted as a graph with a set of disconnected edges, each of which establishes a bijective relation between a substrate and product atom (Fig. 1).

Due to the time consuming nature of manual curation of atom mapping, it is of great importance to have reliable algorithms to predict atom mappings, especially for large sets of reactions found in metabolic databases and genome-scale metabolic network reconstructions. To our knowledge, only the BioPath [1] and KEGG RPAIR [2] databases disseminate manually curated atom mappings. Other metabolic databases such as EC-BLAST [3] MetaCyc [4] and MetRxn [5] include predicted atom mappings.

A genome-scale metabolic reconstruction is a structured knowledge-base that abstracts pertinent information on the biochemical transformations taking place within an organism [6]. Such reconstructions form the basis for the development of condition-specific metabolic models whose functions are simulated and validated by comparison with experimental results. These models are then used in a wide range of biological, biotechnological and biomedical research scenarios. Manual curation reconstructions involves extensive literature review [7] and sometimes sufficient experimental literature is not in existence. This situation has driven the development of a range of software tools that seek to automate parts of the process to generate reconstruction content, e.g., [8]. Recon 3D is the latest human metabolic reconstruction [9], that adds three dimensional metabolite and protein structures to a genome-scale reconstruction for the first time. It is envisaged that this reconstruction and the models derived from it will drive deeper understanding how biochemical processes relate to mechanisms at the atomic scale.

It is fortunate that many atom mapping algorithms have been developed, but which is most suited to predict atom mappings for a genome-scale metabolic network reconstruction? Here, we evaluate six recently published atom mapping algorithms [10–15]. We compare their predictions for more than five thousand metabolic reactions in the latest human metabolic reconstruction, Recon 3D [9]. We also compared these predictions with manually curated atom mappings for a set of 512 human metabolic reactions. Of the manually curated atom mappings, 340 were obtained from the BioPath database [1] and 172 additional reactions were manually curated to ensure that we could compare predictions with representative reaction types from all six top level EC numbers [16] (see Additional file 1: Table 1S). The best performing algorithm was used to predict atom mappings for the latest version of the human metabolic reconstruction, Recon 3D [9].

### Atom mapping algorithms

**Fig. 2:**
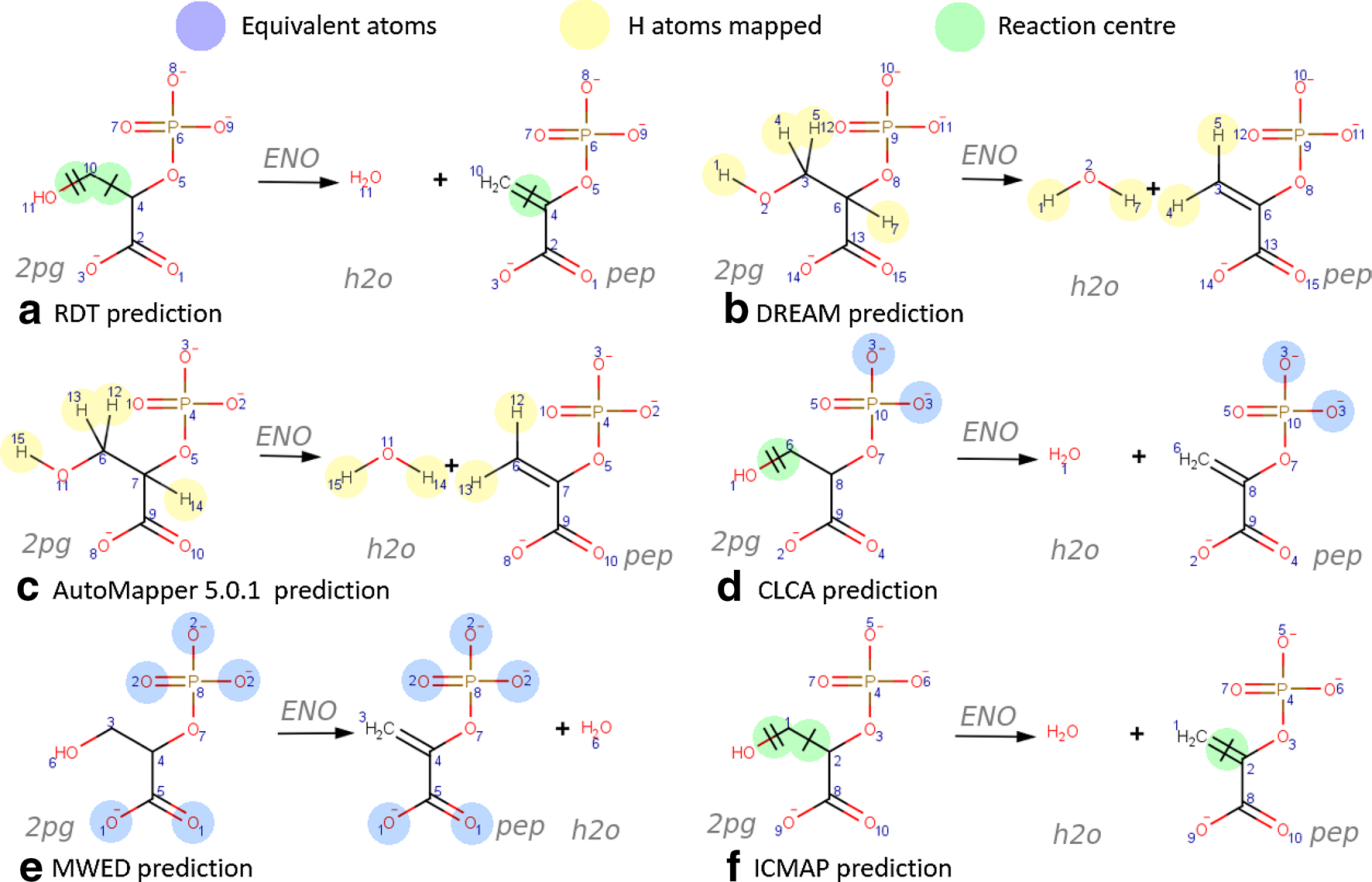
Atom mapping predictions for the enolase reaction. All six compared algorithms returned an accurate atom mapping but included different types of additional information. CLCA and MWED identify equivalent atoms in the reactants (*blue*). DREAM and AutoMapper map hydrogen atoms (*yellow*). RDT, CLCA, ICMAP and MWED all identify reaction centres (*green*). Unlike the other three algorithms, MWED does not identify reaction centres by adding information to the bonds that break and form. Instead, it assigns different colours to the molecular substructures (moieties) that break apart or bind together. The atom mapped reactions are visualised with MarvinView (ChemAxon, Budapest, Hungary), which accepts the RXN and SMILES formats as input

Six academic and commercially available atom mapping algorithms were included in our evaluation *Reaction Decoder Tool* (RDT, [10]), *Determination of Reaction Mechanisms* (DREAM, [11]), *AutoMapper 5.0.1* (AutoMapper, [12], ChemAxon, Budapest, Hungary), *Canonical Labeling for Clique Approximation* (CLCA, [13]), *Minimum Weighted Edit-Distance* (MWED, [14]) within Pathway Tools, and *InfoChem-Map* (ICMAP, [15], InfoChem, Munich, Germany). These algorithms implement different prediction strategies or they use molecular properties, such as the bonds with hydrogen atoms or the use of the stereochemistry to predict atom mapping. They are also equipped with a variable array of advanced features (Fig. 2), including the ability to identify chemically equivalent atoms and reaction centres, as well as the option to map hydrogen atoms. Other distinguishing factors include ease of availability, licensing, and use of standardised data formats. Atom mappings and chemical structure data can be encoded in different atom level chemical reaction formats such as the SMILES [17] or RXN [18] formats. The most useful atom mapping format depends on the quality of the data and the intended application, e.g, the RXN format can hold information about chemical bond changes and stereochemistry. The SMILES format holds a canonical representation of molecules, which is independent of the application used to generate it. Examples of different chemical formats are given in the Additional file 1. A brief description of each atom mapping algorithm follows.

#### DREAM

*D*etermination of *REA*ction *M*echanisms (DREAM) [19] is a Web tool that identifies atom mappings using an optimisation-based approach known as Mixed Integer Linear Optimisation (MILP). This approach aims to minimise the number of bonds broken, bonds formed and bond order changes, between substrates and products. To make predictions it considers chemical properties such as stereochemistry and hydrogen bonding. The functionality of this algorithm is accessible as a web application. With DREAM, it is possible to atom map hydrogen atoms. In practice, the output of the current DREAM implementation does not designate reaction centres or assign chemically equivalent atoms in the output format, although based on the similarity to the MWED algorithm, which is able to carry out both of the aforementioned functions, this should be possible. Atom mappings are predicted from RXN or SMILES files, or a single reaction can be drawn in the web application using the Java Platform, Micro Edition (JME) Molecular Editor. The predicted atom mappings are output as RXN files. Web services and a web user interface for DREAM are available at http://ares.tamu.edu/dream.

#### AutoMapper

AutoMapper [20] (ChemAxon, Budapest, Hungary) uses two approaches to predict atom mapping: maximum common substructure (MCS) and minimum chemical distance (MCD). In MCS, substrate(s) and product(s) are represented as molecular graphs. This approach aims to identify the largest substructures of substrate graphs that are isomorphic to product graphs. For any atom that is not part of an isomorphic substructure, an atom mapping is calculated by MCD, which minimises the number of bonds that are broken and formed. AutoMapper is a tool for atom mapping a single reaction using the desktop application MarvinSketch (ChemAxon, Budapest, Hungary), or multiple reactions using Standardizer (ChemAxon, Budapest, Hungary) via command line. MarvinSketch is available free of charge whereas Standardizer requires a license that is free for academics. Automapper provides the option to map hydrogen atoms, but the tool can neither identify chemically equivalent atoms nor reaction centres. AutoMapper accepts a variety of different chemical formats, including RXN, InChI, and SMILES. It generates atom mappings in RXN or SMILES formats. AutoMapper is available from https://www.chemaxon.com.

#### RDT

Reaction Decoder Tool (RDT) [10] is a Java-based, open-source atom mapping software tool. For each reaction, it returns the best of four atom mappings, predicted with four different algorithms: *Mixture-MCS*, which matches the maximum common substructure between substrates and products; *Min-sub model*, which matches the smallest substructures between the substrates and products; *Max-sub model*, which matches the largest substructures between the substrates and products; and, lastly, *Assimilation model*, which is triggered if a substrate or a product contains a ring system. Once an algorithm has matched a maximal number of atoms, the remaining atoms are mapped according to a similarity score for molecules, and the selection-and-elimination process is repeated until all atoms have been mapped. All four algorithms use the molecule stereochemistry to predict the atom mappings. RDT returns the atom mapping with the minimum number of modified bonds. RDT can be installed on a desktop or accessed via the web application EC-BLAST [3]. RDT can identify the reaction centres but lacks the ability to map hydrogen atoms or identify chemically equivalent atoms. The user is given the choice between RXN and SMILES for both in- and output formats. Web services are available at http://www.ebi.ac.uk/thornton-srv/software/rbl/ and the software at https://github.com/asad/ReactionDecoder.

#### CLCA 

The Canonical Labelling for Clique Approximation [21] (CLCA) algorithm identifies the maximum common substructure between substrates and products using prime factorisation to generate canonical labels for bond-atoms. If a reaction has multiple reactant or product molecular graphs, many combinations of MCS exist. Thus, MCD is used to select a substructure that reduces the number of bond changes between reactants and products. It generates canonical labels using a variety of chemical properties, such as the number of non-hydrogen connections, the number of non-hydrogen bonds, atomic numbers, the sign of charge, the absolute charge, the number of connected hydrogen atoms, the atomic numbers of neighbouring atoms, R or S descriptors for chiral atoms, pro-R or pro-S for prochiral arms, and cis and trans descriptors. CLCA identifies chemically equivalent atoms by using their canonical labels; the algorithm also indicates reaction centres. CLCA can only map hydrogen atoms for reactions with fully protonated molecules. CLCA uses SMILES as its input and output format. The CLCA algorithm is available from https://github.com/maranasgroup/MetRxn/tree/master/Alchemist.

#### MWED 

Similar to DREAM, Minimum Weighted Edit-Distance [22] (MWED) uses an MILP approach that aims to minimise bonds changes. However, this algorithm assigns weights to bonds of the molecules in the reaction, and a specific cost when a bond is modified. The algorithm uses the bonds with hydrogen molecules and the stereochemistry to predict atom mappings. The algorithm is available within the Pathway Tools Software Suite [23], which requires a license that is free of charge for academics. MWED can identify chemically equivalent atoms, as well as reaction centres. The algorithm does not map hydrogen atoms but it does take them into consideration when calculating atom mappings. It supports both RXN and SMILES as input and generates SMILES and MetaCyc output files. Pathway Tools is available from http://biocyc.org/.

#### ICMAP 

InfoChem-Map [15] (ICMAP, InfoChem, Munich, Germany) uses maximum common substructure and minimum chemical distance approaches. It identifies reaction centres using minimum chemical distance when a bond is formed or broken. Unmapped atoms are mapped by minimum chemical distance with two additional chemical rules applied. That is, the breakage and formation of bonds between heteroatoms is given preference over C–C bonds, and bonds with hydrogen atoms are rated the same as C–C bonds. ICMAP is a commercial desktop application. It identifies the reactions centres but cannot identify chemically equivalent atoms or map hydrogen atoms. One extra feature of the ICMAP algorithm is the classification of a reaction regarding the reaction centres indentified using a 15 digit numeric code. Both input and output are in RD file format, which is a container file format for RXN files. ICMAP application is available from http://www.infochem.de/.

## Results

### Prediction accuracy

We evaluated the accuracy of the six atom mapping algorithms (Table 1) by comparing predictions to manually curated atom mappings for 512 reactions (see Methods). The comparison of individual reactions is shown in Additional file 2. Table 1 shows the number of atom mapped reactions that were compared with the manually curated atom mappings.

**Table 1:** Number of evaluated reactions per algorithm

Algorithm	Number of reactions compared	Unmapped reactions
RDT	512	0
DREAM	512	0
AutoMapper	512	0
CLCA	488	24
MWED	477	35
ICMAP	496	16

Due to limited access to some algorithms, we could not predict atom mappings for all 512 manually curated reactions

**Fig. 3:**
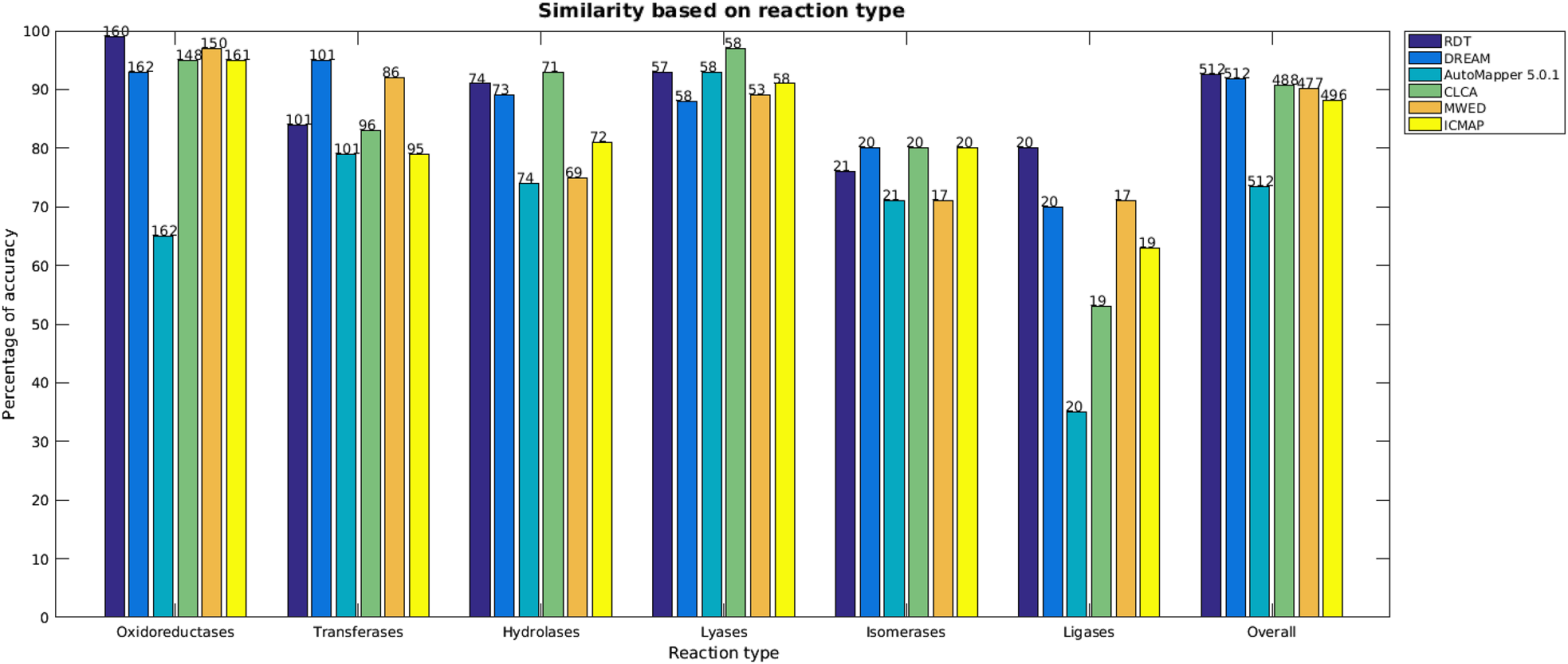
Accuracy by reaction types. Percentage of reactions where predicted atom mappings agreed with the manually curated atom mappings. On each *bar* is shown the number of reactions compared for each algorithm and top level EC number

Metabolic reactions can be classified according to a four digit Enzyme Commission (EC) number assigned to the catalysing enzyme. The first digit, hereafter referred to as the top-level EC number, encodes the type of reaction that the enzyme catalyses (see Additional file 1: Table 1S). The prediction accuracy of the algorithms on representative reactions of all six defined reaction types and the overall accuracy is shown in Fig.  3. Five of the 6 algorithms gave accurate predictions for more than 90% of reactions catalysed by oxidoreductases, with RDT being the most accurate. However, the accuracy of all six algorithms was low for reactions catalysed by ligases. DREAM, CLCA and ICMAP were equally accurate at prediction of atom mappings for isomerases. CLCA was the most accurate for hydrolases and lyases. Finally, DREAM was the most accurate for transferases. The predictions and manual curation for each reaction are given in Additional file 3.

### Additional features

In addition to prediction accuracy, we compared the technical and advanced features of each algorithms. The technical features include the prediction approach, the user interface, the availability, and the file formats used for each algorithm (Table 2). The main advanced features were the ability to identify chemically equivalent atoms and reaction centres, and the option to map hydrogen atoms (Table 3). We also compared additional features, such as the ability to map all atoms in each reaction, the ability to map R groups and the consideration of reactant stereochemistry. Advanced features can be particularly important for certain applications of atom mapping [24–26].

**Table 2:** Comparison of technical features

	Approach	Interface	Availability	Input file formats	Output file formats
RDT	Structure-based	Web and desktop application	Free	RXN, SMILES	RXN, SMILES
DREAM	Optimisation-based	Web application	Free	RXN, SMILES	RXN
MWED	Optimisation-based	Desktop application	Free for academics	RXN, SMILES	SMILES, MetaCyc
CLCA	Structure-based	Algorithm	Free	SMILES	SMILES
ICMAP	Structure-based	Desktop application	Commercial	RXN	RXN
AutoMapper	Structure-based	Desktop application	Free for academics	RXN, SMILES	RXN, SMILES

**Table 3:** Comparison of advanced features

	Equivalent atoms	Hydrogen atoms	Reaction centres	Maps all atoms	Maps R groups	Stereo-chemistry	Maps unbalanced reactions
RDT	$\varvec{\chi }$	$\varvec{\chi }$	$\checkmark$	$\checkmark$	$\checkmark$	$\checkmark$	$\checkmark$
DREAM	$\varvec{\chi }$	$\checkmark$	$\varvec{\chi }$	$\checkmark$	$\checkmark$	$\checkmark$	$\varvec{\chi }$
MWED	$\checkmark$	$\varvec{\chi }$	$\checkmark$	$\checkmark$	$\checkmark$	$\checkmark$	$\varvec{\chi }$
CLCA	$\checkmark$	$\checkmark ^{\rm a}$	$\checkmark$	$\checkmark$	$\checkmark$	$\checkmark$	$\checkmark$
ICMAP	$\varvec{\chi }$	$\varvec{\chi }$	$\checkmark$	$\varvec{\chi }$	$\checkmark$	$\varvec{\chi }$	$\checkmark$
AutoMapper 5.0.1	$\varvec{\chi }$	$\checkmark$	$\varvec{\chi }$	$\checkmark$	$\checkmark$	$\varvec{\chi }$	$\checkmark$

$^{\rm a}$ CLCA can only map hydrogen atoms for reactions with fully protonated molecules

### Application to Recon 3D

**Fig. 4:**
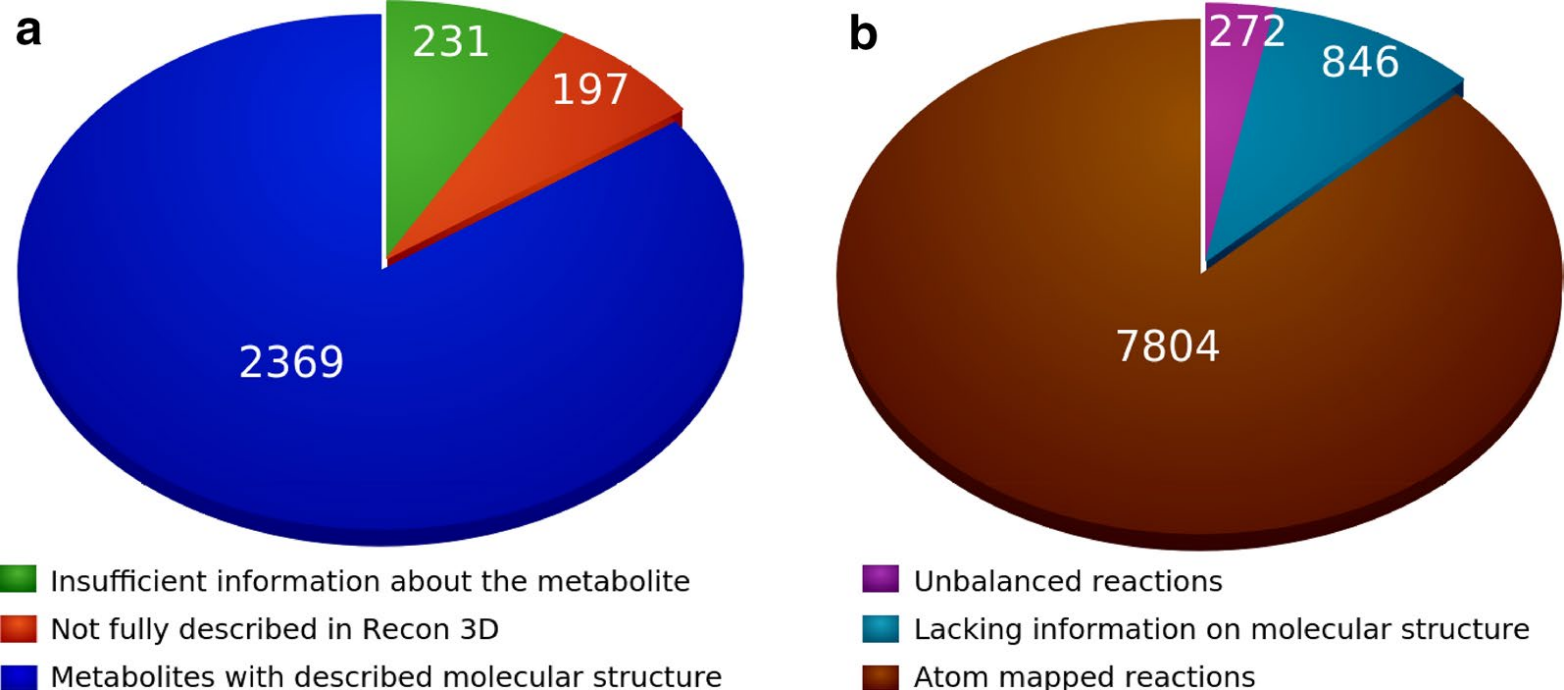
Coverage of metabolites and reactions in Recon 3D. **a** Coverage of unique metabolites structure data. **b** Coverage of reaction atom mapping data

To atom map reactions in a metabolic network reconstruction, one requires chemical structures, reaction stoichiometries, and an atom mapping algorithm. Chemical structures for 2369 (85%) (Fig.  4a) of the 2797 unique metabolites in Recon 3D [9] were obtained were obtained [27] or drawn using information from publicly available sources, such as Recon 2 [28], PubChem [29], Kyoto encyclopedia of genes and genomes (KEGG) [30], Chemical Entities of Biological Interest (ChEBI) [31], Lipid Mass Structure Database (LMSD) [32], BioPath database [33], ChemSpider database [34], and the Human Metabolome DataBase (HMDB) [35]. No chemical structures were obtained for the remaining 428 (15%) unique metabolites due to insufficient information about the precise chemical structure (e.g., eumelanin), or because some Recon 3D reactions do not specify the nature of the reactant sufficiently, e.g., in lipid metabolism, a generic lipid substrate may correspond to a family of compounds, that may differ slightly in structure, due to the number and position of double bonds.

We selected three different algorithms for the atom mapping of mass balanced Recon 3D reactions due to their high accuracy, ease of availability, and predictions without any unmapped atoms. The Reaction Decoder Tool (RDT) was selected to atom map reactions with implicit hydrogen atoms, while DREAM and CLCA were chosen to atom map reaction with explicit hydrogen atoms. Atom mappings were predicted for 7637 mass balanced reactions in Recon 3D, using both RDT and DREAM. Atom mappings were predicted for a further 272 mass imbalanced internal reactions in Recon 3D, using both RDT and CLCA. A remaining 840 internal reactions, that is 10% of all 8748 internal reactions, were not atom mapped due to missing chemical structures (Fig. 4b). Metabolite structures and atom mappings for Recon 3D [9] are disseminated via the Virtual Metabolic Human database (VMH, https://vmh.life/). Metabolite structures are provided in MOL and SMILES formats. Atom mapping data are provided in both RXN and SMILES formats.

## Discussion

The six algorithms compared in this work implement different atom mapping approaches. RDT, AutoMapper 5.0.1, CLCA, and ICMAP implement an approach based on the identification of a common molecular substructure, whereas DREAM and MWED implement an optimisation-based approach [36]. Each algorithm is ideal for different purposes, e.g., the RDT, ICMAP, and MWED algorithms can be used to describe reaction mechanisms as they identify reaction centres. CLCA can be used to enumerate alternative atom mappings by identifying equivalent atoms. DREAM atom maps hydrogen atoms, and can thus be used to identify conserved moieties corresponding to hydrogen atoms in metabolic networks [26] and implemented within the COBRA Toolbox [37]. Finally, AutoMapper has a user-friendly interface and is part of a large suite of useful chemical informatics tools provided by ChemAxon. Due to the accuracy, ease of availability and ability to map all atoms as well as R groups, we chose RDT for atom mapping of Recon 3D reactions with implicit hydrogen atoms, DREAM for its ability to explicitly map hydrogen atoms and CLCA for its ability to map mass imbalanced reactions with explicit hydrogen atoms.

It is especially interesting that five of the six algorithms achieved a prediction accuracy of more than 90%. However, this is somewhat lower than the reported accuracy [21, 22]. This discrepancy may be due to selection of a different set of manually curated atom mappings from the KEGG RPAIR database [2]. Manual curation of more Recon 3D reactions, couple with testing new versions of existing atom mapping algorithms, will, in the future likely lead to prediction accuracy that asymptotically approaches 100%.

If EC numbers were available for all reactions in Recon 3D, a superior strategy would have been to use the prediction of the algorithm with the best accuracy for each top-level EC number. That is, instead of selecting a single algorithm to atom map all reactions in Recon 3D, we could have selected the most accurate algorithm for each reaction type (Fig. 3). However, this was not feasible because a large number of reactions have not yet been assigned an EC number [9, 38]. Moreover, due to the high level of accuracy across all six algorithms, our choice to use RDT was also based on other features such as software availability and accessibility of the user interface.

**Fig. 5:**
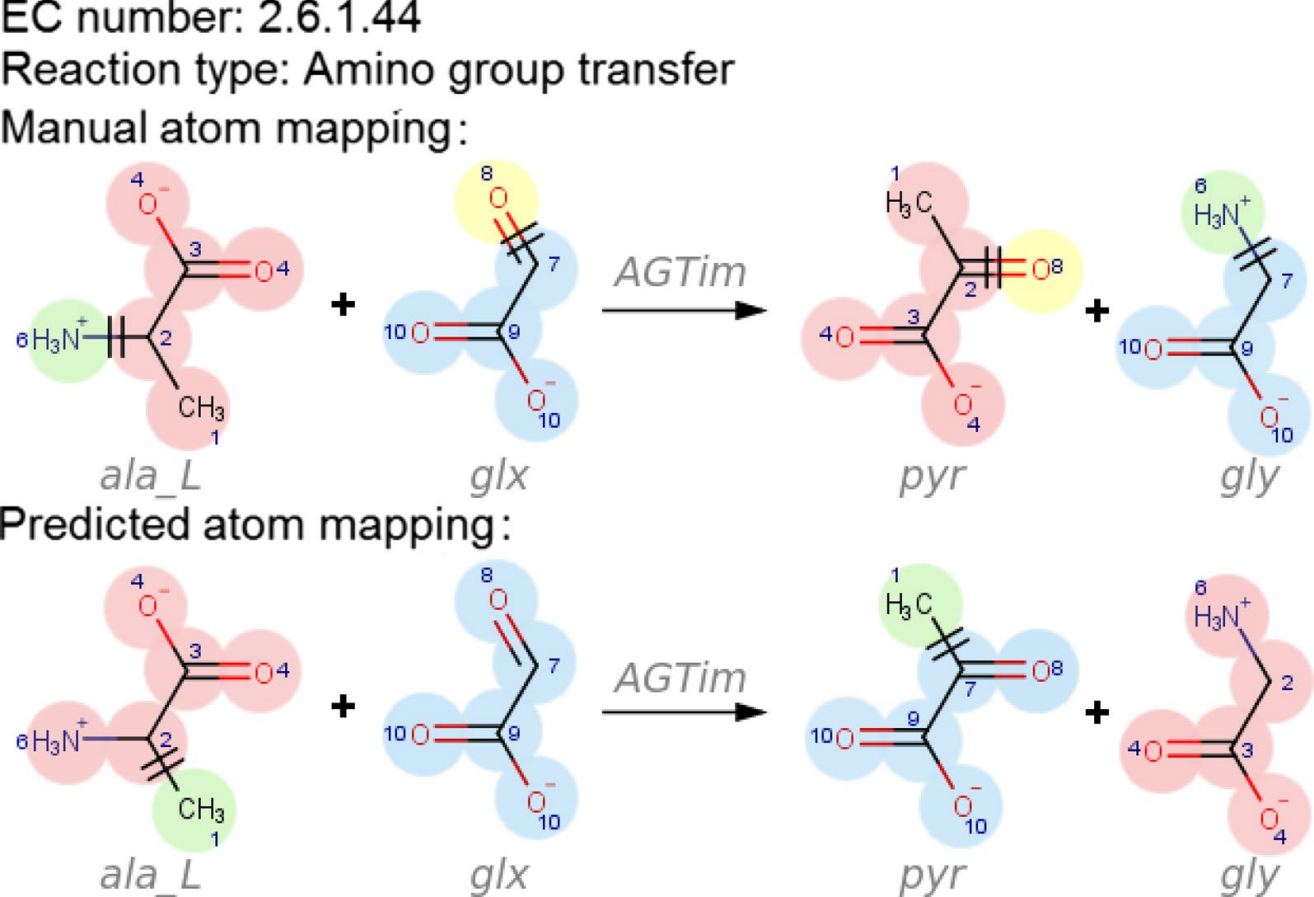
An incorrect reaction mechanism predicted by five algorithms. Alanine-glyoxylate transaminase (VMH ID: AGTim) reaction catalyses the chemical transformation of L-alanine (VMH ID: ala_L) and glyoxylate (VMH ID: glx) into pyruvate (VMH ID: pyr) and glycine (VMH ID: gly). The known reaction mechanism of the alanine-glyoxylate transaminase reaction is represented by the manual atom mapping (*top*). Five algorithms predicted the same incorrect atom mapping for this reaction (*bottom*) [43]. Only the MWED algorithm predicted correctly

**Fig. 6:**
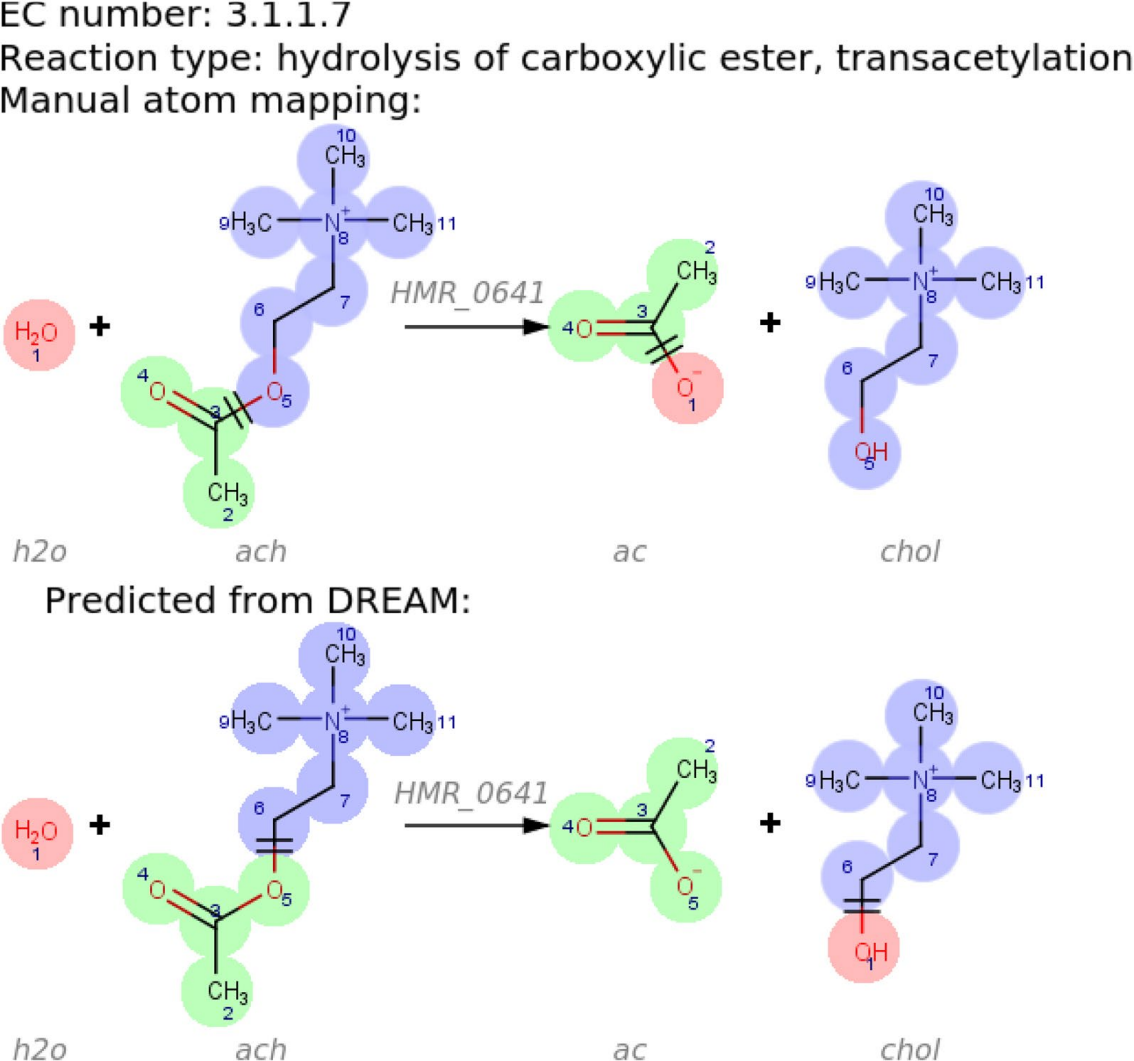
Incorrect addition–elimination mechanism predicted by DREAM. Acetylcholinesterase (VMH ID: HMR_0641) reaction catalyses the breakdown of acetylcholine (VMH ID: ach) and water (VMH ID: h2o) to form acetate (VMH ID: ac) and choline (VMH ID: chol). The predicted mechanism (*bottom*) for the acetylcholinesterase reaction does not correspond to the mechanism described by the EC number (*top*). The (C^3^–O^5^) bond is broken and the (C^3^–O^1^) bond is formed. However, DREAM predicts that the (C^6^–O^5^) bond is broken, followed by formation of the (C^6^–O^1^) bond [43]

The EC number assigned to a reaction contains information about the reaction mechanism, which can be used to identify prediction errors. The most common errors we encountered were the preference for breaking and forming C–C $\sigma$-bonds instead of less stable bond types (Fig. 5), and the incorrect assignment of leaving groups in addition-elimination reactions (Fig. 6). In addition, idiosyncrasies of individual algorithms seem to result in inaccurate predictions for certain types of reactions.

Most algorithms tend to predict atom mappings and thereby reaction mechanisms with the lowest sum total number of bonds that are broken and formed, but often fail to sufficiently penalise the breakage of more stable bonds. This was the case for the alanine-glyoxylate transaminase reaction in Fig. 5, which has the EC number 2.6.1.44. The first number (2) indicates that the type of enzyme that catalyses the reaction is a transferase. The second number (6) indicates that it transfers nitrogen groups. The third number (1) indicates that the nitrogen group is transferred from an alanine molecule. The last number (44) indicates that a nitrogen group is transferred from alanine to glyoxylic acid. In this reaction, five algorithms predicted that the transferred group would be a methyl group, but this is incorrect due to the high energy needed to break a C–C $\sigma$-bond.

Another common error was with prediction of addition-elimination mechanisms. Typically, a nucleophile will attack an electron-deficient centre, which will push electron density towards an adjacent oxygen, followed by electron density being pushed back to the nucleophilic centre, which will eliminate a leaving group. Under acidic conditions, the leaving group is reprotonated to give the resulting alcohol or thiol (see Additional file 1: Figure 1S). Figure 6 shows an example of a prediction error for the acetylcholinesterase reaction. The EC number of the reaction is 3.1.1.7, where (3) indicates a hydrolase reaction, (1) shows that the hydrolysis takes place on an ester bond; more specifically, carboxylic ester hydrolysis (1). The last number (7) indicates hydrolysis of a choline ester. The reaction mechanism predicted by DREAM was not consistent with this EC number.

As expected, DREAM and MWED predictions were similar (Additional file 1: Table 2S) since they are based on a very similar MILP approach. However, although MWED is based on DREAM, the latter obtained greater accuracy when comparing its predictions with the manually-cured atom mappings. Of the 477 predictions that could be compared, the algorithms predictions differed in 69 occasions of which in 30 DREAM predicts correctly and MWED does not, 23 DREAM predicts incorrectly and MWED does not, and in 16 both predictions are wrong. Among the most important differences, MWED fails to correctly assign the leaving groups. Nevertheless, because of the weight, it gives to the bonds, it can correctly predict reaction mechanisms as indicated in Fig. 5.

**Fig. 7:**
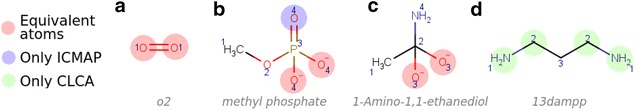
Chemically equivalent atoms. Four molecules with chemically equivalent atoms (*coloured backgrounds*). **a** Molecular oxygen (VMH ID: o2). **b** Methyl phosphate where all three highlighted oxygen atoms are chemically equivalent through resonance. MWED, but not CLCA, can identify the highlighted atoms as being chemically equivalent. **c** 1-Amino-1,1-ethanedio. **d** 1,3-Diaminopropane (VMH ID: 13dampp), which shows that chemically equivalent atoms are not necessarily connected to a shared atom. CLCA, but not MWED, can identify the highlighted atoms as being chemically equivalent

Chemically equivalent atoms in a molecule are atoms that are interchangeable through any symmetric operation (Fig. 7). Reactions of molecules with equivalent atoms have multiple equivalent atom mappings. For instance, all reactions involving molecular oxygen (Fig. 7a) have at least two chemically equivalent atom mappings. A compact representation of all chemically equivalent atom mappings for a single reaction can be achieved by assigning the same atom mapping number to chemically equivalent atoms (Fig. 7). Although the DREAM should, in principle, be able to identify chemically equivalent atoms, only CLCA and MWED assigned chemically equivalent atoms in practice. However, there is room for improvement with both algorithms. CLCA often fails to identify chemically equivalent atoms in resonance structures (Fig. 7b) and MWED fails to identify molecular symmetry (Fig. 7d).

An atom mapping provides an abstract mechanistic description of a chemical reaction. It describes the fate of the atoms and all the bond changes that happen during the reaction. Therefore, when an atom mapping is predicted the reaction centres are identified. RDT, CLCA, MWED, and ICMAP are all able to identify reaction centers and generate their results indicating where the reaction centre is, however, DREAM and AutoMapper do not. The identification of reaction centers is useful for a visual description of the reaction mechanism. These can potentially also be used to predict optimal pathways in a metabolic network, involving the minimum number of bond changes.

**Fig. 8:**
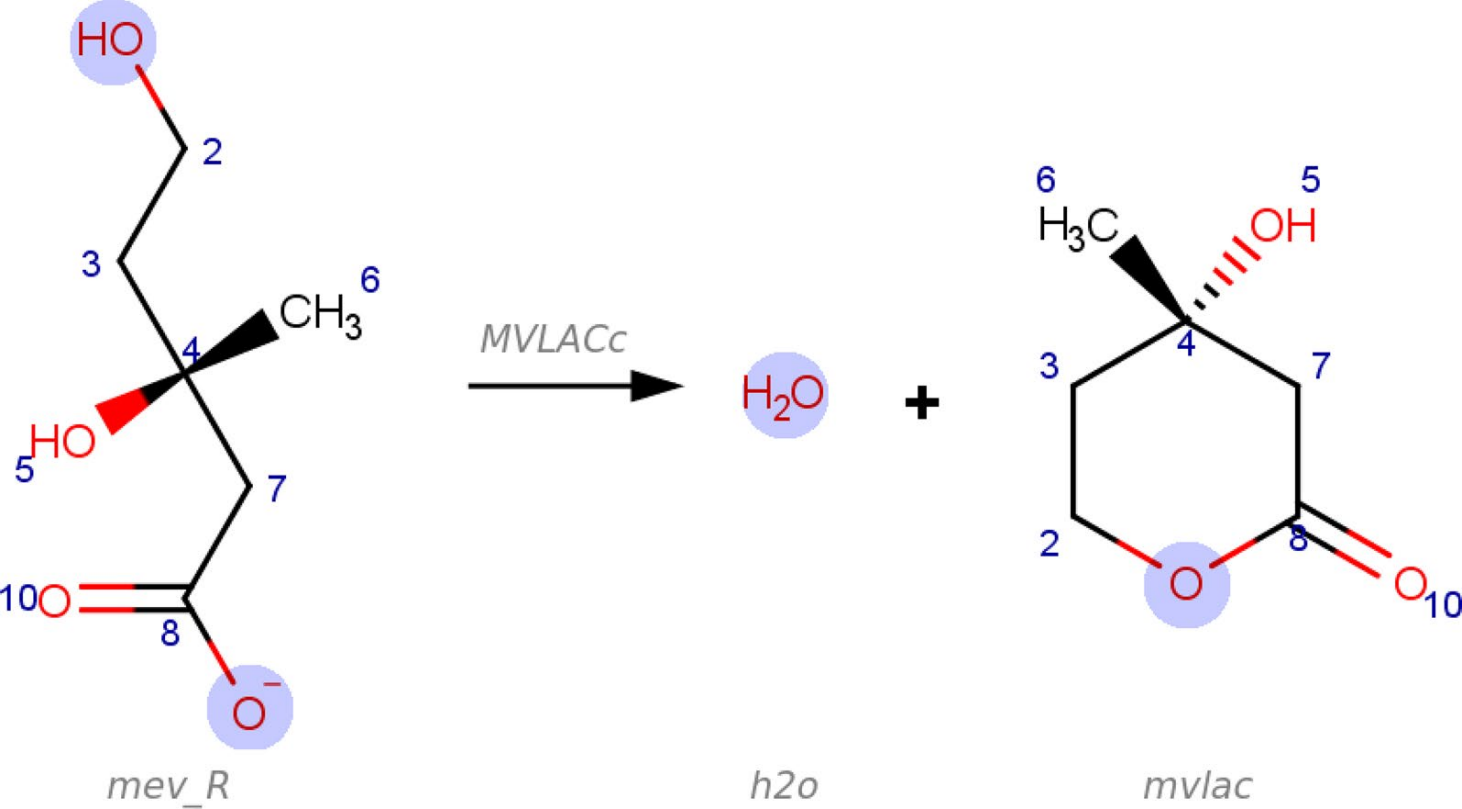
Unmapped atoms. Occasionally, ICMAP leaves some atoms unmapped. In this ICMAP prediction (VMH ID: MVLACc), the oxygen atoms in Mevalonate (VMH ID: mev_R) that are indicated in *blue*, may map to the water molecule (VMH ID: h2o) or the 4-hydroxy-4-methyl-2-oxanone molecule (VMH ID: mvlac)

Two reactions with identical stoichiometry may occur by different reaction mechanisms depending, for example, on the catalysing enzyme. Each reaction mechanism corresponds to a distinct set of atom mappings. The optimisation-based algorithms DREAM and MWED are able to predict multiple optimal atom mappings for a single reaction. On the other hand, ICMAP represent ambiguity in a reaction mechanism by leaving some atoms unmapped. This approach may be useful to represent mechanistic ambiguity in a compact form as a single file (Fig. 8). However, in some cases, it remains unmapped atoms that were not assigned by the MCS process (Fig. 2f, on the left hand side, one oxygen atom in the 2pg molecule and on the right hand side, the h2o molecule).

**Fig. 9:**
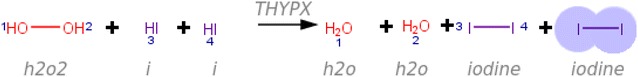
Unbalanced thyroid peroxidase reaction. The thyroid peroxidase reaction (VMH ID: THYPX) catalises the hydrogen peroxide molecules (VMH ID: h2o2) and two hydrogen iodides (VMH ID: i) into two water molecules (VMH ID: h2o) and two molecular iodines (VMH ID: iodine). The reaction can be balanced by adding the unmapped molecular iodine product atoms (*blue background*) on the *left hand side*

The optimisation based algorithms DREAM and MWED require each reaction to be mass elementally balanced in order to assign atom mappings. This requirement is consistent with chemical principles. However, it could be a limitation depending on the objective of the atom mapping. In particular, atom mapping elementally unbalanced reactions could provide an automatic approach to suggest modifications to the reconstruction that could balance the reaction (Fig. 9), or for the automatic assignment of EC numbers [38]. This utility is especially important for reconstructions whose content is at the edge of experimental biochemistry. Nevertheless, for other applications [24, 26, 39] it is necessary to know the fate of all the atoms in the metabolic network.

Atom mapping algorithms use different chemical formats to input and output atom mappings. Each chemical format has unique features that distinguish it from other formats. DREAM, AutoMapper, CLCA, and MWED all used the SMILES format which is a compact string representation of a reaction with canonicalised molecules. RXN files were used by DREAM, MWED, and AutoMapper. This file format can store additional data such as bond changes and stereochemistry. ICMAP was the only algorithm to use the RD format. This format is a concatenation of multiple reactions in RXN format. The developers of MWED have also created their own format based on the data in the MetaCyc database [4]. An additional feature to accept and return at least one standard format (SMILES or RXN) should be a publication criterion for any future atom mapping algorithm. Chemoinformatics applications such as *molConverter* from ChemAxon [20], and OpenBabel [40] can be used to convert from one standard format to another.

Only AutoMapper, CLCA and DREAM provided the option to predict the fate of hydrogen atoms in chemical reactions. RDT, MWED, and ICMAP do not explicitly return the mapping of hydrogen atoms. Nevertheless, bonds involving hydrogen atoms are considered for the assignment of atom mappings by all algorithms, except for AutoMapper v5.0.1. Hydrogen atoms are not as useful as carbon atoms for isotopic labelling in metabolic flux analysis [24], which is currently the main application for atom mapping data. However, as we shall now discuss, there are other applications where this is important.

When reaction stoichiometry is combined with atom mapping data for an entire metabolic network, new applications become possible that are beyond the resolution of reaction stoichiometry alone. For example, given atom mapping data for a stoichiometrically consistent metabolic network, the set of *conserved moieties* can be efficiently computed and identified [26]. Each conserved moiety corresponds to a particular identifiable molecular substructure that is invariant with respect to the chemical transformations of that network. Atom mapping of all atoms is a prerequisite for identification of all conserved moieties. The cardinality of the set of conserved moieties can be enumerated *a priori *as it is equivalent to the row rank deficiency of the corresponding stoichiometric matrix. As such, it is possible to easily check if the expected number of conserved moieties has been computed, given a set of atom mapping data for a stoichiometrically consistent network. In some instances, even with mapping of hydrogen atoms, one or more conserved moiety is not computed with the aforementioned approach [26]. It appears as though mapping of electrons may also be necessary, but the conditions for this requirement remain to be clarified [26]. Every biochemical network will contain at least one conserved moiety corresponding to a hydrogen atom, so this feature is desired for atom mapping of metabolic reconstructions.

The set of conserved moiety vectors forms a sparse non-negative integer basis for the left null space of a stoichiometrically consistent network. Constraints derived from this left null space basis are a fundamental part of kinetic modelling because the amount of each conserved moiety is time-invariant [41]. The set of all conserved moieties for a chemical reaction network give rise to a biochemically intuitive non-negative integer basis for the left nullspace of the corresponding stoichiometric matrix. Of course, one can always compute a linear basis for the left nullspace of a stoichiometric matrix using various linear algebraic algorithms, but then biochemical interpretation of each basis vector is problematic. From this perspective, we advocate for explicit mapping of hydrogen atoms, or at least the option to do so.

Atom mappings are also used to identify the existence and contribution of pathways involved in the metabolism of specific biological molecules by refining carbon flux paths with atomic trace data [25], which can lead to potential biomarkers for diseases. Since conserved moieties consist of a set of atoms that follow the same path through a metabolic network, in principle, it is sufficient to isotopically label a single atom within a moiety to detect the possible paths of that entire moiety through a metabolic network [24]. Additionally, numerical classifiers for enzymes known as Enzyme Commission numbers (EC numbers) [16] can be computationally assigned to reactions in genome-scale metabolic networks [38] using atom mappings. EC numbers establish links between enzymatic reactions, enzymes, enzyme genes, and metabolic pathways. These are just some examples of the many potential applications of atom mapping in genome-scale metabolic networks.

## Conclusions

We focussed on comparing the predictive accuracy of atom mapping algorithms for elementally balanced biochemical reactions with complete structural specification of reactants. Therefore, any conclusions we obtained are specific to this particular atom mapping objective. Many of the algorithms tested have a variety of different advanced features which were not compared in detail so depending on ones objective the optimal algorithmic choice could differ.

Of the six atom mapping algorithms tested for atom mapping of elementally balanced reactions, most had an impressive prediction accuracy of 91% or higher, e.g. the DREAM, CLCA, MWED, ICMAP and RDT algorithms. However different algorithms seem to be more accurate for different types of reaction mechanisms. Selection of an algorithm also depends on factors such as ease of availability of the software, quality of the user interface, and ability to deliver advanced features beyond atom mapping *per se*. Objectively, from the high accuracy achieved by many atom mapping algorithms, one can conclude that atom mapping is an advanced art. To reach perfection, detailed comparison of algorithmic approaches and elucidation of systematic imperfections will be necessary.

From a network perspective, approaching perfection in atom mapping is important because if the probability of an incorrectly mapped atom is $p\in (0,1)$ but the length of a pathway involving that atom is *k* then the probabilty of an incorrectly mapped atom at the end of the pathway is ${\mathcal {O}}(p^{k})$, e.g., $0.91^{10}\cong 0.39$. This is a worst case scenario that assumes the same atom is incorrectly mapped in each of the *k* sequential reactions. Nevertheless, it points out the importance for the atom mapping community of striving for ever higher levels of accuracy.

In our view, this can best be achieved by more detailed comparison of the alternate algorithms, in mathematical form, as well as in their computational implementations, via licensed source code for desktop applications. Expansion of the number of additional features would be valuable. Already, identification of chemically equivalent atoms and reaction centres or mapping of hydrogen atoms are of key importance in certain applications, e.g., identification of conserved moieties and simulation of isotope labelling experiments. The convergence of genome-scale metabolic modelling, chemoinformatics and structural bioinformatics, as illustrated in Recon 3D opens up a host of new applications for atom mapping, not to mention the potential for cross fertilisation of ideas.

## Methods

### Recon 3D

Recon 3D is a genome-scale metabolic reconstruction of human metabolism accounting for ~12,000 metabolic reactions involving ~8000 metabolites [9]. It is not cell-type specific, rather it is an amalgamation of the known metabolic capabilities ocurring in at least one human cell, regardless of type. Recon 3D is the most complete global human network model to date and the first to account for mechanisms at the atomic scale.

### RXN files

After obtaining the chemical structures of the unique metabolites [27], reaction stoichiometries from Recon 3D were used to create the corresponding RXN files using a MATLAB live script (Additional files 4 and 5).

### Manually curated atom mappings

Manually curated atom mappings were obtained from the BioPath database [33] for the 340 Recon 3D reactions that are also on the BioPath database. An additional 196 Recon 3D reactions representative for al 6 top EC-numbers were manually atom mapped according to textbook characterisations of reaction mechanisms [42, 43].

### Algorithms predictions

CLCA, MWED ICMAP and RDT predictions were obtained by contacting the developers of each algorithm. DREAM predictions were obtained by compressing the RXN files obtained into different ZIP files with less than 2 MB of data. Then the ZIP files were uploaded in the DREAM web application. AutoMapper 5.0.1 predictions were obtained by using the ChemAxon application *Standardizer*.

### Evaluation of prediction accuracy

We say that an algorithm accurately predicts the atom mappings for a reaction if each atom mapping for that reaction matches that obtained by manual curation. The accuracy of each algorithm was quantified using the percentage of reactions that were accurately predicted. Predictions that did not match the manually curated atom mappings were double checked manually.

### Standardisation process

Not all algorithms returned results in the same format. In particular, the order of reactants (Fig. 2e, in this example, MWED prediction switched the product molecules) and the order of atoms within reactants varied between algorithms (Fig. 2). Therefore, we standardised the algorithmic output to enable comparison between algorithms and with manually curated data. First, atom mapping predictions in RXN format were converted to SMILES format to obtain the canonical order of atoms in molecules. The conversion was performed using the ChemAxon application *molConverter*. Then, the SMILES strings for substrates and products were sorted by length. If the SMILES for two substrates (or products) were of the same length, they were sorted in alphabetical order. After that, the reactions in SMILES were converted back to RXN format with *molConverter*. Finally, the atom mapping numbers of each atom were re-assigned in ascending order, maintaining the equivalent atoms for the CLCA and MWED algorithms. This standardisation process enabled automatic comparison between predicted and manually curated atom mappings.

#### Unmapped atoms

In some cases, ICMAP did not assign an atom mapping number to all the atoms in a reaction (e.g., oxygen in Fig. 2f). In a post-processing step, atom mapping numbers were automatically assigned to all uniquely identifiable unmapped atoms. A substrate atom was deemed to be uniquely identifiable if it was the only unmapped atom of a particular element and it could therefore only map to one atom of the same element in the products.

#### Chemically equivalent atoms

The identification of chemically equivalent atoms is essential prior to comparing atom mappings. Otherwise, a discrepancy between two equivalent atom mappings would be indistinguishable from a discrepancy due to an incorrect prediction. When algorithms did not identify chemically equivalent atoms, they were identified in a post-processing step using techniques from graph theory implemented in MATLAB (MathWorks, Natick, Massachusetts). Every molecule was represented as a molecular graph $G:=[V,E]$; an ordered pair of vertices, $v\in V$, and edges, $e\in E$. A vertex represents an atom and an undirected edge between two vertices represents a chemical bond. Two atoms *a* and *b* were said to be chemically equivalent if both were of the same element, both were connected to the same atom *c*, and neither was connected to any other atom. The bond type was not considered since equivalent atoms are often part of a resonance structure with delocalised electrons. The chemically equivalent atoms in each substrate were assigned the same atom mapping number. Then, the atom mapping numbers of matched product atoms were updated accordingly. This process was also then repeated in the opposite direction, from products to substrates.

## Additional files

**Additional file 1.** Contains the supplementary information of the manuscript. This includes 1) Different atom mapping chemical formats for reaction cyanase; 2) **Figure 1S**: Ester hydrolysis under basic conditions; 3) **Figure 2S**: Cyanase reaction atom mapped; 4) **Table 1S**: Top level Enzyme Commission number classification; 5) **Table 2S**: Similarity between atom mapping predictions.

**Additional file 2.** Contains the full comparison after the algorithmic and manual check of the of reactions. Full-atom mapping comparison table. With all standardised reactions, RXN file atom identifiers were extracted as an array and processed in MATLAB where they were compared if the reaction was present in the database. Comparisons were made with the following order: 1) cured reactions, 2) DREAM, 3) AutoMapper, 4) CLCA 5) MWED, and 6) ICMAP. In the curated reactions column, there are only two values, 1 or NaN if there was no file. DREAM columncould have 3 values 1 if equal than the curated reactions, 2 if are not equal and NaN if the le does not exist. AutoMapper column has 4 values, 1–3 and NaN. 1 if is equal to curated files, 2 if the mappings are equal to DREAM and 3 if they are not equal to the curated DREAM reactions and NaN if not file existed. So CLCA 1–4 and NaN, MWED 1–5 and NaN, and NaN ICMAP 1–6. If all reactions are equal, all columns 1 values obtained. With the matrix containing all the comparisons similarity of all algorithms was calculated.

**Additional file 3.** Contains two folders, one contains the predictions obtained from each algorithm in RXN format (Folder: algorithmicPredictions), the other contains manually curated atom mappings, (Folder:standardisedPredictions).

**Additional file 4.** A pdf version of atomMappingComparisonScript.mlx.

**Additional file 4.** A MATLAB LiveScript used to standardise the algorithmic predictions, before comparison. Requires MATLAB 2016a and above.

## Abbreviations

RDT: Reaction Decoder Tool
DREAM: Determination of reaction mechanisms
CLCA: Canonical Labelling for Clique Approximation
MWED: Minimum Weighted Edit-Distance
ICMAP: InfoChem-Map
MILP: Mixed Integer Linear Optimisation
MCS: Maximum common substructure
MCD: Minimum chemical distance
EC: Enzyme Commission
