# Supplementary material for: Comparative evaluation of atom mapping algorithms for balanced metabolic reactions: application to Recon 3D
Source: J Cheminform. 2017 Jun 14;9:39. doi: 10.1186/s13321-017-0223-1 (PMC5471289; doi:10.1186/s13321-017-0223-1)
Supplement: Supplementary file 1 — Additional file 1. Contains the supplementary information of the manuscript. This includes 1) Different atom mapping chemical formats for reaction cyanase; 2) Figure 1S: Ester hydrolysis under basic conditions; 3) Figure 2S: Cyanase reaction atom mapped; 4) Table 1S: Top level Enzyme Commission number classification; 5) Table 2S: Similarity between atom mapping predictions. [file 13321_2017_223_MOESM1_ESM.pdf]

Atom mapping formats

Different atom mapping chemical formats are represented for reaction cyanase shown in Figure 2S.

SMILES format

The simplified molecular-input line-entry system (SMILES) is a specification in form of a line notation for describing the structure of chemical species using short ASCII strings. The character "." separates the molecules while the characters "≫" separates the substrates and the products. Different characters are used to indicate the type of bond "=", "#" (double or triple respectively) and the parenthesis indicate the branches of the molecule. Cyanase in SMILES format:

```
[O-:3][C:1]#[N:2].[OH:4][C:5]([OH:6])=[O:7]
>>[NH2:2][C:1]([O-:4])=[O:3].[O:6]=[C:5]=[O:7]
```

MetaCyc format

MetaCyc format consists of 6 lines with the information of the reaction and atom mapping. The first line indicates the MetaCyc reaction identifier. The second line the possible atom mapping for the reaction. The third line the mapping-type which for the moment only NO-HYDROGEN-ENCODING is available. The fourth line indicates the list of compounds on the from-side with their starting and ending indices. In the fifth line is indicated the list of compounds on the to-side with their starting indices. Cyanase in MetaCyc format:

```
REACTION - R524-RXN
NTH-ATOM-MAPPING - 1
MAPPING-TYPE - NO-HYDROGEN-ENCODING
FROM-SIDE - (HCO3 0 3) (CPD-69 4 6)
TO-SIDE - (CARBAMATE 0 3) (CARBON-DIOXIDE 4 6)
INDICES - 4 5 6 3 0 2 1
```

RXN format

RXN files are comprised of a header block where the first line indicates the file format, the second and fourth line usually indicates the name and the reaction stoichiometry respectively, the fifth line indicates the number of molecules in the substrates and products respectively. After the fifth line is contained the MOLs block with the information of the molecules (atom mapping identifier, charge, type of bonds, connectivity, and coordinates). Cyanase in RXN format:

```
$RXN
Cyanase
Mrv1637 081101161707
cyanate + hydrogen carbonate  ammonium + CO2
2 2
$MOL
cyanate
Mrv1637 08111617072D
3 2 0 0 0 0 999 V2000
−3.0580 −0.0893 0.0000 C 0 0 0 0 0 0 0 0 0 1 0 0
−2.2330 −0.0893 0.0000 O 0 5 0 0 0 0 0 0 0 3 0 0
−3.4705  0.6252 0.0000 N 0 0 0 0 0 0 0 0 0 2 0 0
1 2 1 0 0 0 0
1 3 3 0 0 0 0
M CHG 1 2 −1
M END
$MOL
hydrogen carbonate
Mrv1637 08111617072D
4 3 0 0 0 0 999 V2000
−0.0670  0.2232 0.0000 C 0 0 0 0 0 0 0 0 0 5 0 0
 0.6475  0.6357 0.0000 O 0 0 0 0 0 0 0 0 0 4 0 0
−0.7814  0.6357 0.0000 O 0 0 0 0 0 0 0 0 0 6 0 0
−0.0670 −0.6018 0.0000 O 0 0 0 0 0 0 0 0 0 7 0 0
1 2 1 0 0 0 0
1 3 1 0 0 0 0
1 4 2 0 0 0 0
M END
$MOL
ammonium
Mrv1637 08111617072D
4 3 0 0 0 0 999 V2000
 3.5491  0.1786 0.0000 C 0 0 0 0 0 0 0 0 0 1 0 0
 4.2636  0.5911 0.0000 O 0 5 0 0 0 0 0 0 0 4 0 0
 2.8346  0.5911 0.0000 N 0 0 0 0 0 0 0 0 0 2 0 0
 3.5491 −0.6464 0.0000 O 0 0 0 0 0 0 0 0 0 3 0 0
1 2 1 0 0 0 0
1 3 1 0 0 0 0
1 4 2 0 0 0 0
```

M CHG 1 2 −1
M END $
MOL
CO2
Mrv1637 08111617072D
3 2 0 0 0 0 999 V2000
5.6250 0.4241 0.0000 O 0 0 0 0 0 0 0 0 0 6 0 0
6.4500 0.4241 0.0000 C 0 0 0 0 0 0 0 0 0 5 0 0
6.8625 −0.2904 0.0000 O 0 0 0 0 0 0 0 0 0 7 0 0
1 2 2 0 0 0 0
2 3 2 0 0 0 0
M END

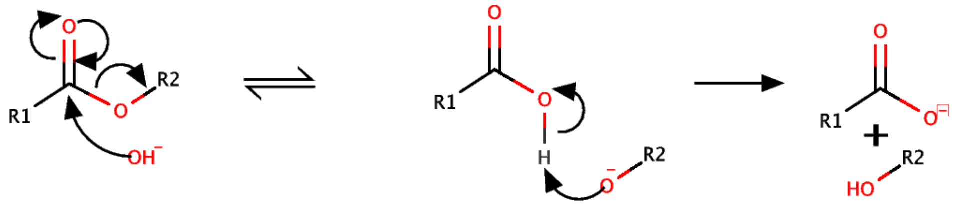

Figure 1: **Ester hydrolysis under basic conditions.** Hydroxide ion reacts at the carbonyl carbon to give a tetrahedral intermediate, from which the alkoxide ion is expelled. The alkoxide ion is reprotonated using the acidic proton on the resulting carboxylic acid.[**?**]

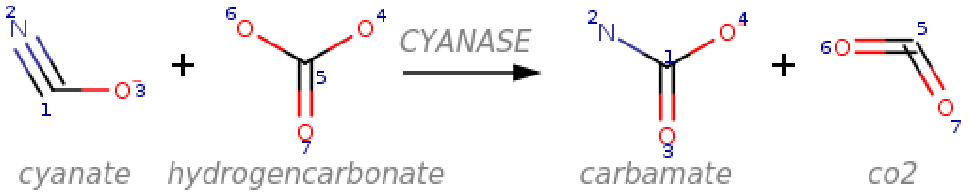

Figure 2: **Cyanase reaction atom mapped.** Cyanase catalyses the reaction of cyanate with bicarbonate to produce carbamate and carbon dioxide.

Table 1: **Top level Enzyme Commission number classification.**

EC	Designation	Function	Reaction example	Enzyme example
**1**	**Oxidoreductases**	Catalyse reduction and oxidation reactions.	AH + B → A + BH (reduction) A + O → AO (oxidation)	Dehydrogenase, oxidase
**2**	**Transferases**	Transfer a functional group.	AB + C → A + BC	Transaminase, kinase
**3**	**Hydrolases**	Catalyse the hydrolysis of various bonds.	AB + H2O → AOH + BH	Lipase, amylase, peptidase
**4**	**Lyases**	Cleave various bonds by means other than hydrolysis and oxidation.	XABY → AB + XY	Decarboxylase
**5**	**Isomerases**	Catalyse isomerization changes within a single molecule.	ABC → BCA	Isomerase, mutase
**6**	**Ligases**	Join two molecules with covalent bonds.	A + B + ATP → AB + ADP + Pi	Synthetase

Table 2: **Similarity between atom mapping predictions.** Pairwise comparisons between the six algorithms and manually curated atom mappings. When comparing each set of atom mappings with the predictions of the algorithms, it can be observed that manually cured reactions have higher similarity than the algorithms predictions.

	Manual	RDT	DREAM	AutoMapper	CLCA	MWED	ICMAP
Manual		93 %	92 %	73 %	91 %	90 %	88 %
RDT	512		47 %	46 %	42 %	54 %	54 %
DREAM	512	3,336		68 %	52 %	74 %	78 %
AutoMapper	512	2,782	3,785		51 %	71 %	71 %
CLCA	488	2,719	3,478	3,014		60 %	61 %
MWED	477	2,858	3,322	2,691	2,876		80 %
ICMAP	496	2,086	2,463	2,261	2,263	2,311	
