# Supplementary material for: Comparative evaluation of atom mapping algorithms for balanced metabolic reactions: application to Recon 3D
Source: J Cheminform. 2017 Jun 14;9:39. doi: 10.1186/s13321-017-0223-1 (PMC5471289; doi:10.1186/s13321-017-0223-1)
Supplement: Supplementary file 4 — Additional file 4. A pdf version of atomMappingComparisonScript.mlx. [file 13321_2017_223_MOESM4_ESM.pdf]

Comparison of atom mapping algorithms

To be able to execute this MATLAB live script, it is necessary to have installed Chemexon license and MATLAB 2016b. The code should be executed in the folder where the MATLAB live script is.

1. Standarize reactions

1.1 Defining directories

```
h = waitbar(0,'Procesing files...');
sandarizerFileDir='~/standarizerFiles';
predictions={'RDT', 'DREAM', 'AutoMapper', 'CLCA', 'MWED', 'ICMAP'};
```

1.2 For each file on each folder standarize the RXN file

```
for k=1:numel(predictions)
    predictionDir=[pwd '/Raw/' predictions{k}];
    fnames=dir([predictionDir '/*.rxn']);
    newDir=[pwd '/standarizedRXN/' predictions{k}];
    mkdir(newDir)
    mkdir([newDir '/notStandarized'])

    for i=1:length(fnames)
        waitbar(i / length(fnames))
        try
            rxn=fnames(i).name;
```

1.3 Convert the RXN file in SMILES format (canonical order)

```
            command = ['molconvert smiles ' [predictionDir '/' rxn] ' -o '  [newDir '/' rxn(1:
            [status,cmdout] = system(command);
```

1.4 Reassigne the order of the molecules in the reactions in the substrates and products (from smaller to bigger)

```
            sortMolecules([newDir '/' rxn(1:end-4) '.smiles'])
            return
```

1.5 Convert SMILES to RXN for the comparison

```
            command = ['molconvert rxn ' [newDir '/' rxn(1:end-4) '.smiles'] ' -o ' [newDir '/
            [status,cmdout] = system(command);
            delete([newDir '/' rxn(1:end-4) '.smiles'])
```

1.6 Asign ascending atom mappings

```
            switch k
                case 4
                    acsendingCLCA([newDir '/' rxn(1:end-4) '.rxn'])
                otherwise
                    acsendingAtomMaps([newDir '/' rxn(1:end-4) '.rxn'])
            end
```

1.7 Calculate equivalent atoms in the molecule

```
            symmetryRXN([newDir '/' rxn(1:end-4) '.rxn'])
        catch
            try
                movefile([predictionDir '/' rxn],[newDir '/notStandarized'])
            catch
            end
        end
    end
end

close(h)
```

2. RXNs comparison

2.1 Load Recon 3D

```
recon3=[pwd '/Recon3.mat'];
load (recon3)
model=modelName;
a=cell({''});
```

2.2 Get the address of all the RXN files to compare

```
prcessedPredictions= ['standarizedRXN/'];
AM_DataBases={'Curated' 'RDT' 'DREAM' 'Chemaxon' 'CLCA' 'MWED' 'ICmap'};
```

2.3 Get all the names of the manually curated files

```
fnames=dir([prcessedPredictions AM_DataBases{1} '/*.rxn']);
```

2.3 Get all the atom mappings for all the files in all folders as arrays

```
for j=1:numel(AM_DataBases)
    for k=1:numel(fnames)
        file=[fullyProcessedDir AM_DataBases{j} '/' char(fnames(k).name)];
        if exist(file, 'file') == 2
            rxnFile = regexp( fileread(file), '\n', 'split')';
            begmol=strmatch('$MOL',rxnFile);
            counter=0;
            for l=1:length(begmol)
                for m=1:str2double(rxnFile{begmol(l)+4}(1:3))
                    counter=counter+1;
                    atoms{counter}=strtrim(rxnFile{begmol(l)+m+4}(61:63));
                end
            end
            eval(sprintf('mappingNumbers(%d).atoms%d=atoms;',k,j));
            clear atoms
        else
            eval(sprintf('mappingNumbers(%d).atoms%d=a;',k,j));
        end
        if j==1
            arrayLength(j)=length(mappingNumbers(j).atoms1);
        end
    end
```

```
end
```

2.4 Convert the obtained arrays into strigs

```
mappingCells = num2cell(cellfun(@(c) [c{:}], permute(struct2cell(mappingNumbers), [3 1 2]), 'U
idz = cellfun('isempty',vertcat(mappingCells{:}));
```

2.5 Comparison of predicted reactions to manually curated reactions (Table)

```
fun = @(x)unique(x,'first');
[~,idx,idy] = cellfun(fun,mappingCells,'UniformOutput',false);
comparisonMatrix = cellfun(@(x,y)x(y),idx,idy,'UniformOutput',false);
comparisonMatrix = vertcat(comparisonMatrix{:});
comparisonMatrix=vec2mat(comparisonMatrix,7);
comparisonMatrix(idz) = NaN;
```

2.6 Calculate percentage of similarity of predictions and manually curated reactions

```
for j=2:numel(AM_DataBases)
    totalRxn=find(~isnan(comparisonMatrix(:,j)));
    correctRxn=find(comparisonMatrix(totalRxn,j)==1);
    percentages(j-1)=(length(correctRxn)/length(totalRxn))*100;
end
```

2.7 Create figure of comparison

```
figure(1)
bar(percentages)
title('Accuracy of predictions')
xlabel('Algorithms')
ylabel('Percentage of accuracy')
set(gcf,'color','w');
ax = gca;
ax.XTick = 1:6;
ax.XTickLabel = {'RDT', 'DREAM','AutoMapper','CLCA','MWED', 'ICMAP'};
```

2.8 Accuracy per atoms

```
D = comparisonMatrix-1;
D(bsxfun(@or,idz,idz(:,1))) = NaN;
m=D;
for i=1:length(mappingNumbers)
    V(i)=length(mappingNumbers(i).atoms1);
end
m(isnan(m))=0;
AMcorrect=100-sum(m)./sum(repmat(V.',1,7).*isfinite(D))*100
```

3. Accuracy by reaction type

3.1 Use table created in 2.5 and data in Recon 3D to calculate the accuracy per reaction type

```
totalEC=zeros(6,7);
correctEC=totalEC;
for i=1:numel(fnames)
    file=fnames(i).name;
    ecNumber = model.rxnECNumbers{strmatch(file(1:end-4), model.rxns,'exact')};
    if ~isempty(ecNumber)
        index=regexp(ecNumber,'\d');
        totalEC(str2double(ecNumber(index(1))),:)= totalEC(str2double(ecNumber(index(1))),:)+~
        correctEC(str2double(ecNumber(index(1))),:)= correctEC(str2double(ecNumber(index(1))),
    end
end
percentageMatrixEC=round(correctEC./totalEC*100);
percentageMatrixEC(:,1)=[];
percentageMatrixEC(7,:)=percentages;
totalEC(:,1)=[];
totalEC(7,:)=[512 512 512 488 477 496];
```

3.2 Create accuracy per reaction type figure

```
figure(3)
hb = bar(percentageMatrixEC);
set(gcf,'color','w');
title('Similarity based on reaction type')
legend('RDT', 'DREAM','AutoMapper 5.0.1','CLCA','MWED', 'ICMAP','location','northeastoutside')
xlabel('Reaction type')
ylabel('Percentage of accuracy')
ax = gca;
ax.XTick = 1:7;
ax.XTickLabel = {'Oxidoreductases','Transferases','Hydrolases','Lyases', 'Isomerases', 'Ligase

barWidth = hb.BarWidth;
numCol = size(percentageMatrixEC,2);
cnt = 0;
for ii = totalEC'
    cnt = cnt + 1;
    xPos = linspace(cnt - barWidth/2, cnt + barWidth / 2, numCol+1);
    idx = 1;
    for jj = xPos(1:end-1)
        val = totalEC(cnt,idx);
        y = percentageMatrixEC(cnt,idx);
        text(jj, y + 1, num2str(val));
        idx = idx +1;
    end
end
```

4. Comparison of all predicted reactions

4.1 Obtain all the reactions

```
predictionsDirs={[prcessedPredictions AM_DataBases{1}]};
for i = 2:numel(AM_DataBases)
   predictionsDirs{i}=[prcessedPredictions AM_DataBases{i}];
end
for k = 1:numel(predictionsDirs)
   fnames = dir(fullfile(predictionsDirs{k}, '/*.rxn')) ; % fullfile for cross-OS compatibilit
   rxns{k} = {fnames.name};
end
rxns = unique([rxns{:}])';
```

```

```

4.2 Assign EC number if possible

```
for j=1:length(rxns)
    comparison(j).rxn=rxns{j}(1:end-4);
    ecNumber= strmatch(comparison(j).rxn, model.rxns,'exact');
    comparison(j).ECnumber=modelName.rxnECNumbers(ecNumber(1));
end
```

4.3 Reads the atom mappings for all the RXN file in all the directories and save them as strings

```
a=cell({''});
for i=1:length(AM_DataBases)
    for j=1:length(rxns)
        file=[fullyProcessedDir AM_DataBases{i} '/' rxns{j}];
        if exist(file, 'file') == 2
            rxnFile = regexp( fileread(file), '\n', 'split')';
            begmol=strmatch('$MOL',rxnFile);
            counter=0;
            for k=1:length(begmol)
                for l=1:str2double(rxnFile{begmol(k)+4}(1:3))
                    counter=counter+1;
                    atoms{counter}=strtrim(rxnFile{begmol(k)+l+4}(61:63));
                end
            end
            eval(sprintf('mappingNumbers(%d).atoms%d=atoms;',j,i));
            clear atoms
        else
            eval(sprintf('mappingNumbers(%d).atoms%d=a;',j,i));
        end
        if i==2
            arrayLength(j)=length(mappingNumbers(j).atoms2);
        end
    end
end
```

4.4 Is created a matrix for the comparisons where colums represent the algorithms and rows atom mapped reactions

```
mappingCells = num2cell(cellfun(@(c) [c{:}], permute(struct2cell(mappingNumbers), [3 1 2]), 'U
idz = cellfun('isempty',vertcat(mappingCells{:}));
```

4.5 Comparison of all predictions

```
fun = @(x)unique(x,'first');
[~,idx,idy] = cellfun(fun,mappingCells,'UniformOutput',false);
comparisonMatrix = cellfun(@(x,y)x(y),idx,idy,'UniformOutput',false);
comparisonMatrix = vertcat(comparisonMatrix{:});
comparisonMatrix=vec2mat(comparisonMatrix,6);
comparisonMatrix(idz) = NaN;
for i=1:length(comparison)
    for j=1:6
        eval(sprintf('comparison(%d).DB%d=comparisonMatrix(%d,%d);',i,j,i,j));
    end
```

```
end
```

4.6 The perecentage of simmilarity of each of the algorithms (upper triangular) and the times compared (lower triangular) are calculated

```
a = permute(comparisonMatrix,[3,2,1]);
b = permute(comparisonMatrix,[2,3,1]);
accuracyRxn = sum(bsxfun(@eq,a,b),3);
totalRxn = sum(~isnan(bsxfun(@plus,a,b)),3);
out = tril(totalRxn,-1) + round(triu(accuracyRxn./totalRxn'*100,1))
```

Local functions

sortMolecules

```
function sortMolecules(filename)

%   The the molecules in the substras and products in the SMILES are sorted by size
%
%   sortMolecules(filename)
%
%   INPUT
%   filename                        Name of the SMILES file.
%
%   OUTPUT
%   Overwrite a SMILES file with sorted molecules
%
%   By:     German Andres Preciat Gonzalez
%   Date:   11/11/2015

smilesFile = fileread(filename);
formula=strsplit(strtrim(smilesFile),'>>');

newSmiles=[];
for i=1:2
    molecules=strsplit(strtrim(formula{i}),'.');
    sort(:,1)=cellfun(@(x)length(x),molecules)';
    [l a]=size(sort);
    sort(:,2)=1:l;
    sort=sortrows(sort);
    for j=1:l
        if j==l
            if i==1
                newSmiles=[newSmiles molecules{sort(j,2)} '>>'];
            else
                newSmiles=[newSmiles molecules{sort(j,2)}];
            end
        else
            newSmiles=[newSmiles molecules{sort(j,2)} '.'];
        end
    end
    clear sort
end
fid2 = fopen(filename, 'w');
fprintf(fid2,'%s\n',newSmiles);
fclose(fid2);
```

```
end
return
```

acsendingAtomMaps

```
function acsendingAtomMaps(filename)

%   The atom mappings of predictions are calculated ascendantly
%
%   acsendingAtomMaps(filename)
%
%   INPUT
%   filename                        Name of the rxn file.
%
%   OUTPUT
%   Overwrite a .rxn with ascendantly atom mappings
%
%   By:     German Andres Preciat Gonzalez
%   Date:   11/11/2015

rxnFile = regexp( fileread(filename), '\n', 'split')';
substrates=str2double(rxnFile{5}(1:3));             % Extracts the # of molecules (substractes
products=str2double(rxnFile{5}(4:6));
begmol=strmatch('$MOL',rxnFile);

noOfAtoms=0;
for i=1:substrates
    for k=1:str2double(rxnFile{begmol(i)+4}(1:3)) % number of atoms in the molecule
        noOfAtoms=noOfAtoms+1;
        if str2double(rxnFile{begmol(i)+k+4}(61:63))~=0
            oldMapNum(noOfAtoms)=str2double(rxnFile{begmol(i)+k+4}(61:63));
            rxnFile{begmol(i)+k+4}(61:63)='   ';
            rxnFile{begmol(i)+k+4}(61+3-length(num2str(noOfAtoms)):63)=num2str(noOfAtoms);
        end
    end
end

for i=substrates+1:substrates+products
    for k=1:str2double(rxnFile{begmol(i)+4}(1:3))
        atomMapNum=str2double(rxnFile{begmol(i)+k+4}(61:63));
        if atomMapNum~=0
            atomMatrixIndex=find(oldMapNum==atomMapNum);
            if ~isempty(atomMatrixIndex)
                rxnFile{begmol(i)+k+4}(61:63)='   ';
                rxnFile{begmol(i)+k+4}(61+3-length(num2str(atomMatrixIndex(1))):63)=num2str(at
                atomMatrixIndex(1)=[];
            else
                atomMatrixIndex=999;
                rxnFile{begmol(i)+k+4}(61:63)='   ';
                rxnFile{begmol(i)+k+4}(61+3-length(num2str(atomMatrixIndex(1))):63)=num2str(at
            end
        end
    end
end

fid2 = fopen(filename, 'w');
fprintf(fid2, '%s\n', rxnFile{:});
fclose(fid2);
```

acsendingAtomMaps

```
function acsendingCLCA(filename)

%   The atom mappings of CLCA predictions are calculated ascendantly
%
%   acsendingCLCA(filename)
%
%   INPUT
%   filename                        Name of the rxn file.
%
%   OUTPUT
%   Overwrite a .rxn with ascendantly atom mappings
%
%   By:     German Andres Preciat Gonzalez
%   Date:   11/11/2015

rxnFile = regexp( fileread(filename), '\n', 'split')';

substrates=str2double(rxnFile{5}(1:3));
products=str2double(rxnFile{5}(4:6));

begmol=strmatch('$MOL',rxnFile);

% create a matrix with previous and acending values
atom=0;
for i=1:substrates
    for k=1:str2double(rxnFile{begmol(i)+4}(1:3)); % number of atoms in the molecule
        atom=atom+1;
        atomMapNum(atom,1)=str2double(rxnFile{begmol(i)+k+4}(61:63));
        atomMapNum(atom,2)=atom;
    end
end

% reasign equivalent atoms
for i=1:atom
    indexes=find(atomMapNum(:,1)==atomMapNum(i,1));
    for j=1:length(indexes)
        if indexes(j)~=i
            atomMapNum(indexes(j),2)=atomMapNum(indexes(1),2);
        end
    end
    if atomMapNum(i,1)==0
        atomMapNum(i,2)=0;
    end
end

%   asign in RXN file
% products
c=0;
for i=1:substrates
    for k=1:str2double(rxnFile{begmol(i)+4}(1:3)); % number of atoms in the molecule
        c=c+1;
        rxnFile{begmol(i)+k+4}(61:63)='   ';
        rxnFile{begmol(i)+k+4}(61+3-length(num2str(atomMapNum(c,2))):63)=num2str(atomMapNum(c,
    end
end

% substrates
```

```
for i=substrates+1:substrates+products
    for k=1:str2double(rxnFile{begmol(i)+4}(1:3)); % number of atoms in the molecule
        newMap=find(atomMapNum(:,1)==str2double(rxnFile{begmol(i)+k+4}(61:63)));
        rxnFile{begmol(i)+k+4}(61:63)='   ';
        rxnFile{begmol(i)+k+4}(61+3-length(num2str(atomMapNum(newMap(1),2))):63)=num2str(atomM
    end
end

fid2 = fopen(filename, 'w');
fprintf(fid2, '%s\n', rxnFile{:});
fclose(fid2);
```

Symmetry

```
function symmetryRXN(filename,rxnFilePath)
%   Calculate the chemically equivalent atoms in a reactions
%
%   symmetryRXN(filename,rxnFilePath)
%
%   INPUT
%   filename                        Name of the rxn file.
%   rxnFilePath                     Path of the rxn file (optional).
%
%   OUTPUT
%   Overwrite a .rxn file with the chemically equivalent atoms calculated problem solve.
%
%   By:     German Andres Preciat Gonzalez
%   Date:   11/11/2015

if ~exist('rxnFilePath','var')                              %
    rxnFilePath=[pwd filesep];                              %
end                                                         % If there is not path the program

cd(rxnFilePath)
rxnFile = regexp( fileread(filename), '\n', 'split')';
Substrates=str2double(rxnFile{5}(1:3));             % Extracts the # of molecules (substractes
Products=str2double(rxnFile{5}(4:6));
begmol=strmatch('$MOL',rxnFile);

for i=1:Substrates
    numberOfAtoms=str2double(rxnFile{begmol(i)+4}(1:3));
    numberOfBonds=str2double(rxnFile{begmol(i)+4}(4:6));
    if numberOfBonds~=0
        if numberOfBonds==1
            repaceMatrix(1)=str2double(rxnFile{begmol(i)+5}(61:63));
            repaceMatrix(2)=str2double(rxnFile{begmol(i)+6}(61:63));
            rxnFile{begmol(i)+6}(61:63)='   ';
            rxnFile{begmol(i)+6}(61+3-length(num2str(repaceMatrix(1))):63)=num2str(repaceMatri
            for j=Substrates+1:Substrates+Products
                for k=1:str2double(rxnFile{begmol(j)+4}(1:3))
                    if str2double(rxnFile{begmol(j)+4+k}(61:63))==repaceMatrix(2)
                    rxnFile{begmol(j)+k+4}(61:63)='   ';
                    rxnFile{begmol(j)+k+4}(61+3-length(num2str(repaceMatrix(1))):63)=num2str(r
                    end
                end
            end
        else
            for j=1:numberOfBonds
               bondmatrix(j,1)=str2double(rxnFile{begmol(i)+4+numberOfAtoms+j}(1:3));
```

```
                bondmatrix(j,2)=str2double(rxnFile{begmol(i)+4+numberOfAtoms+j}(4:6));
            end
            % find sigle atom graphs
            posSimAt=find(hist(bondmatrix(:)',unique(bondmatrix(:)'))==1);
            % find with which atom they are conected
            posSimAt(2,:)=zeros(1,length(posSimAt));
            savePos=zeros(1,length(posSimAt));
            for j=1:length(posSimAt)
                [x,y]=find(bondmatrix==posSimAt(1,j));
                savePos=x;
                [x,y]=find(bondmatrix(savePos,:)~=posSimAt(1,j));
                posSimAt(2,j)=bondmatrix(savePos,y);
            end
            a=unique(posSimAt(2,:)');
            if length(a)~=1
                c=find(hist(posSimAt(2,:)',a')>1);
                vector=zeros(1,length(c));
                for j=1:length(c)
                    vector(j)=a(c(j));
                end
            else
                vector=a;
            end

            count=1;
            for j=1:length(vector)
                for k=1:length(posSimAt)
                    if posSimAt(2,k)==vector(j)
                        vtc(count)=posSimAt(1,k);
                        count=count+1;
                    end
                end
                for k=1:length(vtc)
                    atMatrix(k,2)=vtc(k);
                    atMatrix(k,1)=str2double(rxnFile{begmol(i)+4+vtc(k)}(61:63));
                end
                atMatrix = sortrows(atMatrix);
                vtc=atMatrix(:,2);
                    if length(vtc)~=1
                        if isequal(rxnFile{begmol(i)+4+vtc(1)}(32:33),rxnFile{begmol(i)+4+vtc(
                        numToBeAdd=str2double(rxnFile{begmol(i)+4+vtc(1)}(61:63));
                        for k=2:length(vtc)
                            numToBeCh=str2double(rxnFile{begmol(i)+4+vtc(k)}(61:63));
                            rxnFile{begmol(i)+4+vtc(k)}(61:63)='   ';
                            rxnFile{begmol(i)+4+vtc(k)}(61+3-length(num2str(numToBeAdd)):63)=n

                            for l=begmol(Substrates+1):length(rxnFile)
                                products=strsplit(strtrim(rxnFile{l}));
                                if length(rxnFile{l})==69 && str2double(rxnFile{l}(61:63))==nu
                                    rxnFile{l}(61:63)='   ';
                                    rxnFile{l}(61+3-length(num2str(numToBeAdd)):63)=num2str(nu
                                end
                            end
                        end
                        end
                    end
                clear vtc atMatrix
                count=1;
            end
        end
    clear posSimAt bondmatrix vector a c
```

```
    end
end

% Assign chemically equivalent atoms in products
for i=1+Substrates:Substrates+Products
    numberOfAtoms=str2double(rxnFile{begmol(i)+4}(1:3));
    numberOfBonds=str2double(rxnFile{begmol(i)+4}(4:6));
    if numberOfBonds~=0
        if numberOfBonds==1
            repaceMatrix(1)=str2double(rxnFile{begmol(i)+5}(61:63));
            repaceMatrix(2)=str2double(rxnFile{begmol(i)+6}(61:63));
            rxnFile{begmol(i)+6}(61:63)='   ';
            rxnFile{begmol(i)+6}(61+3-length(num2str(repaceMatrix(1))):63)=num2str(repaceMatri
            for j=Substrates+1:Substrates+Products
                for k=1:str2double(rxnFile{begmol(j)+4}(1:3))
                    if str2double(rxnFile{begmol(j)+4+k}(61:63))==repaceMatrix(2)
                    rxnFile{begmol(j)+k+4}(61:63)='   ';
                    rxnFile{begmol(j)+k+4}(61+3-length(num2str(repaceMatrix(1))):63)=num2str(r
                    end
                end
            end
        else
            for j=1:numberOfBonds
               bondmatrix(j,1)=str2double(rxnFile{begmol(i)+4+numberOfAtoms+j}(1:3));
               bondmatrix(j,2)=str2double(rxnFile{begmol(i)+4+numberOfAtoms+j}(4:6));
            end
            % find sigle atom graphs
            posSimAt=find(hist(bondmatrix(:)',unique(bondmatrix(:)'))==1);
            % find with which atom they are conected
            posSimAt(2,:)=zeros(1,length(posSimAt));
            savePos=zeros(1,length(posSimAt));
            for j=1:length(posSimAt)
                [x,y]=find(bondmatrix==posSimAt(1,j));
                savePos=x;
                [x,y]=find(bondmatrix(savePos,:)~=posSimAt(1,j));
                posSimAt(2,j)=bondmatrix(savePos,y);
            end
            a=unique(posSimAt(2,:)');
            if length(a)~=1
                c=find(hist(posSimAt(2,:)',a')>1);
                vector=zeros(1,length(c));
                for j=1:length(c)
                    vector(j)=a(c(j));
                end
            else
                vector=a;
            end

            count=1;
            for j=1:length(vector)
                for k=1:length(posSimAt)
                    if posSimAt(2,k)==vector(j)
                        vtc(count)=posSimAt(1,k);
                        count=count+1;
                    end
                end
                for k=1:length(vtc)
                    atMatrix(k,2)=vtc(k);
                    atMatrix(k,1)=str2double(rxnFile{begmol(i)+4+vtc(k)}(61:63));
                end
                atMatrix = sortrows(atMatrix);
```

```
                vtc=atMatrix(:,2);
                    if length(vtc)~=1
                        if isequal(rxnFile{begmol(i)+4+vtc(1)}(32:33),rxnFile{begmol(i)+4+vtc(
                        numToBeAdd=str2double(rxnFile{begmol(i)+4+vtc(1)}(61:63));
                        for k=1:length(vtc)
                            numToBeCh=str2double(rxnFile{begmol(i)+4+vtc(k)}(61:63));
                            rxnFile{begmol(i)+4+vtc(k)}(61:63)='   ';
                            rxnFile{begmol(i)+4+vtc(k)}(61+3-length(num2str(numToBeAdd)):63)=n

                            for l=1:begmol(Products+1)
                                products=strsplit(strtrim(rxnFile{l}));
                                if length(rxnFile{l})==69 && str2double(rxnFile{l}(61:63))==nu
                                    rxnFile{l}(61:63)='   ';
                                    rxnFile{l}(61+3-length(num2str(numToBeAdd)):63)=num2str(nu
                                end
                            end
                        end
                        end
                    end
               % end
                clear vtc atMatrix
                count=1;
            end
        end
    clear posSimAt bondmatrix vector a c
    end
end

fid2 = fopen(filename, 'w');
for row = 1:length(rxnFile)
    fprintf(fid2,'%s\n',rxnFile{row});
end
   fclose(fid2);
```
